# Supplementary material for: Neurological outcomes and predictive factors in traumatic spinal cord injury patients in the intensive care unit
Source: PLoS One. 2025 May 29;20(5):e0323433. doi: 10.1371/journal.pone.0323433 (PMC12121827; doi:10.1371/journal.pone.0323433)
Supplement: Attachment — Submitted filename: Response to Reviewers.docx [file pone.0323433.s003.docx]

**Response to Reviewers**

**Manuscript ID: PONE-D-25-08324R1**

Title: Neurological Outcomes and Predictive Factors in Traumatic Spinal Cord Injury Patients in the Intensive Care Unit

Dear Academic Editor and Reviewers,

We sincerely appreciate the reviewers' positive feedback and the opportunity to further improve our manuscript. We have carefully addressed all the comments and suggestions as outlined below:

**Reviewer #1:**

1. Add the effect size for your multivariate analysis so we can determine how much stronger that early surgery and blood pressure management can be to determine neurological recovery.

**Response:** We have added effect size data in the Results section and in Table 7, including standard errors (SE) for each covariate. Specifically, we report the odds ratios (OR), 95% confidence intervals (CI), and SEs from the multivariate logistic regression model, which demonstrate the strength of association between early surgical intervention and targeted blood pressure management. These findings suggest that both early hemodynamic optimization and timely surgical intervention are independently associated with improved neurological recovery. For instance, early surgery (OR = 2.841, 95% CI: 1.088–7.419, SE = 0.490) and early blood pressure management (OR = 2.296, 95% CI: 1.036–5.086, SE = 0.406) were found to be significant predictors of favorable neurological outcomes.

2. Can you adjust the title of Table 7 to clearly identify your adjustment?

**Response:** We have revised the title of Table 7 to: "Multivariate Logistic Regression Analysis of Postoperative Neurological Improvement in Traumatic SCI Patients (Adjusted for age, gender, ISS, injury level/severity, steroid use, mannitol use, combined injury, and surgical approach)."

3. For your limitation section, can you add references and elaborate more on the limitations of the ASIA scale?

**Response:** We have expanded the Limitations section to include a more detailed discussion of the limitations of the ASIA scale and added relevant references to support these points. We also clarified in the Methods section that patients classified as ASIA grade E were excluded due to the lack of neurological deficits, with minimal risk of functional deterioration significant enough for inclusion. However, we acknowledge the concern that some of these patients could deteriorate, and this limitation has now been explicitly noted in the revised Limitations section.

4. Did you consider any questionnaire for autonomic recovery as well? It will be important to elaborate on this.

**Response:** We acknowledge this important aspect. We have now added a sentence in the Discussion noting that autonomic recovery was not assessed using standardized questionnaires such as the Spinal Cord Independence Measure (SCIM)  or the International Standards to document remaining Autonomic Function after Spinal Cord Injury (ISAFSCI) , which is a limitation of our study and should be addressed in future research.

5. From your record, what is the shape of spinal fracture that was associated with the best neurological recovery, and what was the type of spinal fracture that was associated with the worst recovery?

**Response:** We appreciate the reviewer’s interest in the association between spinal fracture morphology and neurological recovery. However, our current dataset did not include sufficient detail on fracture shape classification to allow for a reliable analysis of fracture patterns and their correlation with outcomes. Therefore, we are unable to provide conclusive observations regarding which fracture types were associated with the best or worst neurological recovery. We have clarified this in the Discussion section and suggested it as an important area for future research, which we plan to investigate in subsequent studies with dedicated radiographic data collection and classification. We are particularly interested in exploring how specific fracture morphologies may influence spinal cord damage and recovery trajectories in different clinical scenarios.

6. Please clearly identify in the abstract that you used the ASIA scale as a measure of neurological recovery.

**Response:** This has been clearly stated in the Methods portion of the Abstract: “Neurological recovery was assessed using the American Spinal Injury Association (ASIA) impairment scale.”

**Reviewer #2: (No additional comments provided.)**

**Reviewer #3:**

1. Abstract Result section: 'early surgery (≥24h)' should be corrected to 'early surgery (≤24h).'

**Response:** Thank you for identifying this. We have corrected this typo in both the Abstract and the main Results section.

2. Also in the 'Treatment of TSCI and Status at Discharge' section, ‘early surgery (≥24h)’ should be ‘early surgery (≤24h).’

**Response:** This error has been corrected.

3. Please check and edit any other typos in the paper.

**Response:** We have conducted a thorough proofreading of the entire manuscript and corrected typographical and grammatical errors to improve readability and precision.

We appreciate the thoughtful and constructive feedback from the reviewers and editor.

We believe that the revised manuscript has been significantly improved and hope it now meets the criteria for publication in PLOS ONE.

Sincerely,

Weiting Chen, M.D. Corresponding Author
